# Benign or disordered development? Assessment and simulation of security of highly aggregated tourist crowds in China

**DOI:** 10.1371/journal.pone.0240547

**Published:** 2020-10-29

**Authors:** Jie Yin, Yahua Bi

**Affiliations:** 1 College of Tourism, Huaqiao University, Quanzhou, China; 2 Department of Tourism and Convention, Pusan National University, Busan, Republic of Korea; Qatar University, QATAR

## Abstract

Arising with increasing security issues in highly aggregated tourist crowds (HATCs), widespread attention has been dedicated to security status. Assessing and forecasting the security status of HATCs in various situations related to tourist destinations is an important strategy of security management. Thus, this study constructed a system dynamic flow diagram for the security evaluation of HATCs. The relevant data were collected on perceptions of crowded tourists through questionnaires at Tianyou Peak during China's National Day (Golden Week Holiday). Additionally, efforts were made to conduct online surveys at Shanghai Disney Park and Shilin Night Market in Taipei, since crowding always occurs in these two areas. Empirical results based on Vensim software suggest that HATC status is the result of the coupling of various influencing factors and the result of the benign coupling of the three subsystems: multi-source pressure, state variation, and management response. HATC security presents a changing trend of “increase-decrease-recovery”. Differences exist in the changes of HATC security status in different spaces and at different time nodes. The findings also indicated that HATCs that appear in the daytime are more stable than HATCs that appear at special time nodes. This study highlighted that the security management of HATCs should focus on systematization, differentiation, and precision management.

## 1. Introduction

Modern society is becoming more crowded, and the incidents caused by it are increasing [1–3]. Due to the negative impacts on security, satisfaction, and loyalty, crowding has received growing research attention [4–7]. With economic development, the number of tourists has grown rapidly, leading to complex crowding issues [6,8]. Especially in China, due to the popularity of tourism activities and the concentration of tourist time [9], a large number of tourists easily gather at the entrances/exits of scenic areas, transfer stations in scenic areas, popular scenic spots, ticket windows, and tourist centers, forming highly aggregated tourist crowds (HATCs). Zeitz et al. [10] argued that the terms "crowd" and "mass gathering" usually are used interchangeably in previous studies, which were defined as an organized event occurring in a defined space with many people attending [11]. Concerning the definition of tourists, they are net consumers of economic resources within the regions visited [12]. Combining the definition of crowd and tourists, we argue that the HATC is a unique and dense crowd of more than 50 tourists in a special space with a density higher than 2 people/m^2^ [13–15]. HATCs have become a special tourist group for tourism research [16,17].

HATCs and overcrowding issues can induce various negative effects, such as water pollution [18], environmental destruction [19], and service facility destruction [20]. Crowded and dense environments can cause confusion and frustration [4]. Furthermore, overcrowding can even lead to conflicts in society [21] and threaten tourists’ safety [8]. Hence, HATCs have become an important part of destination security management [22] and have presented enormous challenges for emergency preparedness [23]. Concerning crowd management, it is effective to introduce management interventions before congestion [24]. Management interventions can become a prerequisite for a clear understanding of what security status HATCs have. As such, HATC security becomes a consideration for destination development [25]. Due to the spatial attributes of crowd management, previous research has focused on crowds in different environments, such as mountains [26], beaches [27,28], underwater environments [29], urban cities [7,16,17,30], heritage sites [31,32], national parks [21,33–35], theme parks [36], festival sites [37], and cruise ships [4,38]. However, previous research on crowd management was criticized as being limited to a single environment and lacking mutual comparison. Efforts to compare with different situations can contribute to early warning and security management, which is essential because risks appear in various situations and should be treated differently.

To bridge the research gaps, we explored the security assessment and forecasting of HATCs in different regions and provided references for the security management of HATCs. Specifically, this paper endeavors to address three issues. First, we assess and predict the security status of HATCs. Yin et al. [39] qualitatively proposed the occurrence mechanism and coping paths of incidents of HATCs while failed to examine, evaluate, and forecast the safety status of HATCs in different situations. The HATC, as a form of the crowd, is a living system [3,40]. Hence, it is necessary to quantitatively assess and forecast HATC security status for management interventions. Second, by comparing the changes of HATC security in different environments, this study provides rich strategies for security management. Scholars suggest that comparing the security status of different scenarios is critical to HATC management [39]. Therefore, this paper compared and analyzed HATC security in three different places including a mountain, theme park, and night market, which helps to effectively manage HATCs in different situations. Third, we figured out the time nodes for management interventions according to the assessment and simulation results of HATC security status. Early management interventions can affect the tourists’ experience, while late management interventions may be unable to effectively manage HATCs. This research effectively presents the appropriate management intervention time.

The significance of this research can make the following contributions to the existing literature. First, this study employed the system dynamics method to dynamically assess and simulate HATC security status, with an emphasis on the objective evaluation of HATCs rather than subjective crowding perception. Additionally, this study quantitatively confirmed the occurrence mechanism and coping paths of incidents of HATCs proposed previously, which is the further verification for existing research. Second, this paper evaluated and simulated the security status in different situations, broadening our understanding of HATCs, and overcoming the limitations of focusing on one certain environment. Due to the spatial attributes of crowd management, it is important to explore the changes and differences in HATC security. Theoretically, by focusing on the objective security evaluation and simulation of HATCs and comparing the security status in different situations, we can enrich the security knowledge on HATCs. Practically, it is beneficial for crowd management to assess the security status and forecast changes in HATC security status. Specifically, we revealed the time for adopting management interventions by indicating the early warning times for HATCs in different places, which is very useful for managing the HATCs. Discovering the time node for adopting management interventions can contribute to risk management.

The rest of the paper proceeds as follows. Section 2 displays the relevant literature review. Section 3 presents the research design including the introduction of the method, study sites, and data collection. Section 4 introduces the assessment and simulation results of HATCs in different environments. Section 5 illustrates the conclusions, discussion, implications, and limitations of this research.

## 2. Literature review

### 2.1 Tourist crowding

As tourism activities and the number of tourists increase, crowding, as a general phenomenon, has become increasingly frequent. As a result of physical and perceptual impacts on the tourism industry [35], tourist crowding has received widespread concern because it can negatively affect destinations. Previous research on tourist crowding mainly has focused on three issues.

First, scholars noted that the perception of crowding varies in different situations [41,42]. From the time and space factor aspect, tourists would feel higher crowding in hot and dry weather rather than in cool weather [41]. Tourists with higher accessibility to tourism resources would have lower crowding perceptions [42]. Additionally, tourists’ perceived crowding can be affected by environmental factors [43,44]. Heywood and Murdock [43] found that when a large number of tourists stay in a small-size tourism attraction, they would have stronger crowding perceptions. Tourists’ improper behaviors can lead to tourists’ crowding perceptions as well. For instance, uncivilized behaviors including littering and environmental pollution may make people feel more crowded [44].

Second, scholars have noticed the negative effects of tourist crowding [45,46]. For example, previous research mentioned that the more tourists there are in an area, the lower the acceptability levels of tourists [47]. Besides, crowding has a negative impact on the residential quality, visitor experience [7], perceived luxury brand value [4], and satisfaction [26,31].

Third, a number of studies presented that crowding is affected by multiple factors, such as individual preferences [30], tourists’ socio-demographic characteristics, socio-behavioral factors [48], nationality [32], tourism motivation [9], age, and gender [26]. Perceived crowding is a subjective evaluation of the surroundings [49] and is therefore affected by the number or density of visitors [29]. Additionally, queuing and waiting for mass gatherings have a significant negative effect on perceived crowding [26]. Furthermore, overcrowding may reduce the safety of tourists [50] and cause numerous problems in tourism security [8].

### 2.2 Tourist security

In view of the negative impacts of tourist crowding, tourist security has become an increasing concern to the tourism industry [51,52]. Coupled with the importance of tourism prosperity [53,54], numerous studies have been conducted on risk, safety, and security issues for different types of tourists, such as adventure travelers [55], self-driving tourists [56], international tourists [57,58], international women travelers [59], domestic tourists [60], self-guided tourists [61], backpackers [62,63], and senior tourists [64]. With the normalization and popularization of tourism activities, HATCs appear more and more frequently in destinations, especially in the most populous country in the world—China. Due to a large number of tourists and the high density of this special tourist group, it is difficult to manage effectively. Nevertheless, tourist security has not received the attention it deserves [3,25,65].

### 2.3 HATC security

To date, the research on HATC security is still in the initial stage. Previous studies are relatively few and focus on the following two aspects. On the one hand, many studies pay attention to the factors that affect HATC security. HATC is a complex dynamic system, including the three subsystems: the pressure subsystem, state subsystem, and response subsystem [15]. HATC security is affected by multiple factors such as people, facilities, environment, and management [13]. HATC density is an essential factor, and the higher the density, the higher the probability of an incident [25,66]. When HATC density is higher than 5 people/m^2^, the crowd will be stagnant and incidents will be prone to occur [67]. On the other hand, existing research reveals safety management strategies of HATCs, particularly on congestion mitigation [68,69], dynamic adjustment of traveling routes [70], and evacuation strategies [71–73]. Evaluating the HATC security status is a prerequisite for risk management. Researchers utilize system dynamics to assess and simulate HATC security status only in one specific situation [3]. It is worth noting that the security management of HATCs should focus on spatial attributes. By evaluating the security status in different environments, destination management can make plans according to different situations to overcome the issues caused by HATCs.

## 3. Research design

### 3.1 Method

#### System dynamics

The dense crowd is a complex dynamic system [74]. Previous research suggests that a crowd accident was induced by the collapse of the dynamic system [25]. HATCs are a special dense crowd that can be regarded as a dynamic system [3]. The occurrence of HATC incidents is the result of multiple-factor interactions [75]. Hence, it is necessary to clarify the interactions of factors influencing HATC security before evaluation and simulation. Forrester (1961) [77] proposed system dynamics to analyze enterprise problems such as production management and inventory management. Based on the relationship between systems and internal mechanisms, system dynamics can assess complex systems and behaviors through the establishment and manipulation of models [76–78]. The system needs to have factors that determine the function of a system [77]. According to interactions of factors, we can determine the causal relationships. By dynamically simulating changes in the system, we can evaluate the security [76,78–81] and provide early security warning [82]. As such, this study employed system dynamics to simulate the HATC security level.

#### Coupling analysis

Interactions of systems can form a coupling, and the coupling degree refers to the extent of these interactions [83,84]. We can use the coupling to understand systems coordination because coupling indicates the process of a system moving from a chaotic one to a calm one [83,84]. If the systems interact with each other well, then the coupling coordination degree has a good status. Improper management of a HATC may create an unsafe environment for tourists [39]. Namely, when the coupling coordination degree of this system is poor, the system is in a dangerous environment [3]. We use the coupling coordination degree to judge the security level of the system. According to the multiple system coupling coordination principles, this research devised a multiple system coupling coordination function based on the system coupling degree model. The coordination coupling degree is examined as follows.

$$D={\left(C\times T\right)}^{1/2},\text{T}=a_{1}\times {\text{t}}_{1}+a_{2}\times {\text{t}}_{2}\ldots a_{m}\times {\text{t}}_{m}$$ (1)

t_1_, t_2_, and t_m_ refer to each system of the coordinated coupling model. *a*_1_, *a*_2_, and *a*_m_ are the weights of each system. D is the coordination coupling degree. C is the coupling degree acquired by formula (2). Additionally, *m* represents the number of systems and equals 3 in current research.

$$C=m{\left[({\text{t}}_{1}\times {\text{t}}_{2}\times {\text{t}}_{3}\ldots \times {\text{t}}_{\text{m}})/(\prod_{i\neq j,ij=1,2\ldots m}\left({\text{t}}_{1}{\text{+t}}_{2}\text{+}\ldots {\text{t}}_{\text{m}}\right))\right]}^{1/m}$$ (2)

This research utilized system dynamics and coupled analysis to evaluate and simulate the HATC security level (Table 1). According to Wang et al (2019) [98]’s classification criteria for coupling coordination types, this research classified security and warning levels as follows: we divided interval value (0, 0.4) into highly dangerous (HD), interval value (0.4, 0.7) into moderately dangerous (MD), interval value (0.7, 0.9) into low dangerous (LD), and interval value (>0.9) into slightly dangerous (SD).

**Table 1:** Security and warning level of HATCs.

HATC Security	Security Level	Early Warning Level
[0, 0.1]-S10	Highly Dangerous (HD)	Serious Warning: Red Warning (R)
(0.1, 0.2]-S9		
(0.2, 0.3]-S8		
(0.3, 0.4]-S7		
(0.4, 0.5]-S6	Moderately Dangerous (MD)	Moderate Warning: Orange Warning (O)
(0.5, 0.6]-S5		
(0.6, 0.7]-S4	Low Dangerous (LD)	Slight Warning: Yellow Warning (Y)
(0.7, 0.8]-S3		
(0.8, 0.9]-S2	Slightly Dangerous (SD)	Maintain the Status (M)
(0.9, 1]-S1		

### 3.2 The security assessment model of HATCs

**Fig 1:**
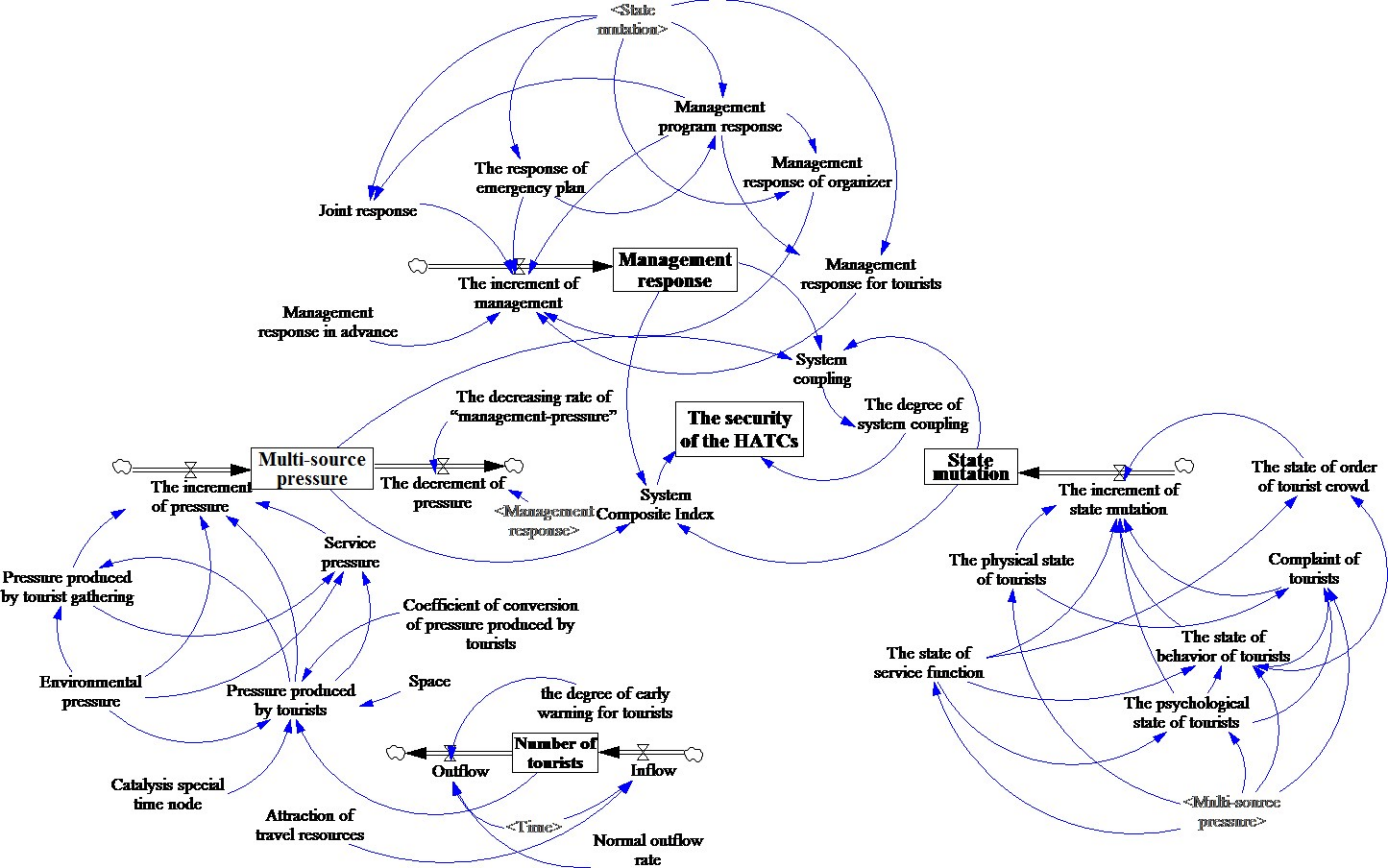
The system dynamics model to assess HATCs security.

Security exists in HATC status. Weak system status in a crowd may cause incidents. Therefore, the status level of a system infers the security level of a system to a certain extent. Since HATC status was affected by subsystem interactions, this paper assessed the level of interaction effects to estimate the HATC security level. Based on the previous findings [3,15,39,75], 23 interacting factors influence HATC security. This paper employed system dynamics to analyze interaction relations of these 23 factors. Additionally, this research added auxiliary variable factors to clearly explain HATC security and constructed a system dynamics model to examine HATC security (see Fig 1).

### 3.3 Study site

Yin et al. [3] analyzed 264 cases on HATC incidents and found that HATCs can easily appear in mountainous areas, theme parks, and traditional cultural streets. According to the previous results [3], this paper selected three typical environments, namely Tianyou Peak scenic area, Shanghai Disneyland Park, and Shilin Night Market in Taipei, to investigate HATC security status.

#### Mountain: Tianyou Peak scenic area, Wuyi Mountain

Wuyi Mountain is a famous mountain destination in China, receiving more than 2 million tourists every year, and Tianyou Peak is an important part of Wuyi Mountain and the main place for tourists. According to the maximum daily and temporary carrying capacity of 5A Scenic Spots announced by the China National Tourism Administration in July 2015, the maximum carrying capacity of the main scenic spot in Wuyi Mountain is 35,000 people and the temporary carrying capacity in Tianyou Peak is 15,000 people. In the past five years, Wuyi Mountain has received more than 180,000 tourists on China's National Day. It also received more than 22,000 tourists every day. Hence, HATCs are easy to appear in Tianyou Peak during China's National Day. As such, this study used Tianyou Peak as the research object to understand HATCs in a mountainous area.

#### Theme Park: Shanghai Disneyland Park

As the first Disneyland Park in mainland China, Shanghai Disneyland attracted 4 million visitors in four months after its opening in June 2016. As of October 31, 2017, the number of visitors to this theme park has exceeded 27 million. In the two years since its opening, Shanghai Disneyland has served more than 34 million visitors with 46,575 average daily visitors. HATCs are often formed in Shanghai Disneyland Park. Scholars mentioned that crowding is especially common during peak periods around the most popular rides in Shanghai Disneyland [85]. Therefore, this study selected Shanghai Disneyland Park to investigate HATC security status.

#### Traditional Cultural Street: Shilin Night Market in Taipei

As a specifically Taiwanese cultural and nightlife phenomenon and one of the three most popular attractions in Taiwan [86], Shilin Night Market attracts lots of tourists [87]. In Shilin Night Market, crowded roads, blocked traffic, extremely high crowd density, and many tourists appear frequently. Due to a large number of tourists who choose to go to Shilin Night Market, HATCs are easily formed, along with the factors related to night environments mentioned above, and local night market restrictions. However, due to the complex nighttime environment, such as traffic and crowd movement, Shilin Night Market poses a high level of security issues for HATCs. Therefore, this article regards Shilin Night Market as a research site for HATCs.

### 3.4 Questionnaire design and data collection

Based on 23 interacting factors influencing HATC security, this study designed a questionnaire with 34 items (Appendix A). The periods when HATCs appeared are different, so we investigated three study sites at different periods. According to the number of tourists appearing at Tianyou Peak during China's National Day in the past and related crowding reports, HATCs that appeared in this time period are more representative. This paper conducted a field questionnaire survey at Tianyou Peak during China's National Day in 2017 (To be noticed, according to judgment of the on-site investigation and related statistics, there are HATCs in Tianyou Peak scenic area, during the National Day golden week (7 days) in 2017, Tianyou Peak scenic area of Wuyi Mountain received 228,100 tourists, an average of 28,513 tourists per day, which exceeded the instantaneous capacity of Tianyou Peak scenic area, which are 15,000 people. Therefore, we argued that there are HATCs in Tianyou Peak scenic area). As mentioned previously, HATCs often occur in the Shanghai Disneyland Park and the Shilin Night Market. Therefore, this study does not strictly limit the specific time nodes and methods for the investigation in the two situations. Shanghai Disneyland Park attracts a large number of visitors during the day, while Shilin Night Market has many visitors at night. In terms of the time when HATCs appeared, this study conducted a survey of HATCs in Shanghai Disneyland during the day and a survey in Shilin Night Market at night. We collected data using online questionnaires in these two places. A total of 300 questionnaires were distributed and 281 were recovered, with a recovery rate of 93.67%; 266 questionnaires are valid, with an effectiveness rate of 88.67% at Tianyou Peak. We received 256 valid questionnaires from Shanghai Disneyland and 269 valid questionnaires from Shilin Night Market. The raw data obtained from questionnaires were used for the security assessment of the initial period of HATCs and was used as the initial value for the security simulation. It should be noted that the factors and variables (Table 2) involved in the system dynamics model (Fig 1) are assessed by tourists in study sites according to their experiences. Thus, the initial values of the variables shown in Table 2 are assigned as the average of all the interviewers’ assessments.

**Table 2:** Weight of observed items.

Variables	Items	Weight			Variables	Items	Weight		
		AHP	Entropy	Average			AHP	Entropy	Average
Pressure produced by tourists	Q2	0.3750	0.1400	0.258	Management response of organizer	Q25	0.4831	0.2275	0.355
	Q3	0.3750	0.3235	0.349		Q26	0.0931	0.2607	0.177
	Q4	0.1250	0.1953	0.160		Q27	0.2119	0.2427	0.227
	Q5	0.1250	0.3411	0.233		Q28	0.2119	0.2691	0.241
Environmental pressure	Q8	0.2000	0.2532	0.227	The response of emergency plan	Q29	0.5	0.5042	0.502
	Q9	0.6000	0.2959	0.448		Q30	0.5	0.4959	0.498
	Q10	0.2000	0.4509	0.325	Joint response	Q32	0.4286	0.3015	0.365
The state of service function	Q15	0.1667	0.4886	0.328		Q33	0.1429	0.3119	0.227
	Q17	0.8333	0.5114	0.672		Q34	0.4286	0.3866	0.408
The physical state of tourists	Q20	0.2000	0.3019	0.251					
	Q21	0.6000	0.3616	0.481					
	Q22	0.2000	0.3365	0.268					

## 4. Assessment and simulation of HATC security

### 4.1 Assessment process

#### The initial value of the variables

We determined the variables of the system dynamics model for assessing HATC security based on data obtained from the questionnaires. It should be noted that some variables need to be calculated by observed variables, so the weight of each observed variable needs to be determined. This paper employed the Analytic Hierarchy Process (AHP method) and the Entropy Method to analyze and calculate the weight of variable parts in the assessment model regarding the average weight of the AHP Method and the Entropy Method for the final weights. Analytic Hierarchy Process [88] is developed to resolve the complex problems, which may consist of multiple-criteria, multiple-levels, complex structure. A criteria weighing method (AHP) calculates the weight by a pairwise comparison using a nine-point scale [88,89]. Various studies employed the AHP method to calculate the weight of factors [90,91]. Additionally, the Entropy Method is an effective method to accurately weigh the relative importance of the identified criteria [92,93]. The base of the Entropy Method is the volume of information used to calculate the index’s weight [94], which is widely applied to evaluate the weight [95,96].

The AHP analysis was completed by three professors who were respectively familiar with the Tianyou scenic area, Shanghai Disneyland Park, and Shilin Night Market. The weights of variables were shown in Table 2. Besides, the initial value of other variables was directly assigned.

Some variables, such as pressure increments, and pressure decrements, cannot be directly calculated from observed variables. These variables need to be calculated by other variables of the assessment model. This study employed the AHP method to calculate them by the weight of the influencing factors. The weights of these variables are shown in the Table 3.

**Table 3:** The weight relations among variables and their assessment model influencing factors.

Variables	Influencing factors	Weight	Variables	Influencing factors	Weight
The state of order of the tourist crowd	The state of the behavior of tourists	0.8333	Management response of organizers	State mutation	0.2500
	The state of service function	0.1667		Management program response	0.7500
Pressure	The pressure produced by tourists	0.5538	The state of the behavior of tourists	Multi-source pressure	0.1031
	Service pressure	0.1259		The psychological state of tourists	0.5258
	The pressure produced by tourist gathering	0.0727		Tourists Complaint	0.1297
	Environmental pressure	0.2477		The state of service function	0.2414
Joint response	State mutation	0.8750	Management response for tourists	State mutation	0.3333
	Management program response	0.1250		Management program response	0.6667
The psychological state of tourists	The state of service function	0.6667	The pressure produced by tourist gathering	Environmental pressure	0.5
	Multi-source pressure	0.3333		The pressure produced by tourists	0.5
Service pressure	The pressure produced by tourists	0.2583	Complaint of tourists	Multi-source pressure	0.1047
	The pressure produced by tourist gathering	0.1047		The psychological state of tourists	0.2583
	Environmental pressure	0.6370		The physical state of tourists (check state or status)	0.6370
Status Mutation	The state of order of tourist crowd	0.3005	Management	Joint response	0.1193
	The state of service function	0.0448		Management response for tourists	0.3204
	The psychological status of tourists	0.1105		The response of an emergency plan	0.0614
	The state of the behavior of tourists	0.3272		Management response of organizers	0.2943
	Tourists Complaint	0.0845		The response of an emergency plan	0.1063
	The physical state of tourists	0.1327		Management response in advance	0.0983
The pressure produced by tourists	(number of tourists/the area of the space)*Coefficient of conversion of pressure produced by tourists	0.5936	Management response of organizers	State mutation	0.2500
	Environmental pressure	0.2493		Management program response	0.7500
	Catalysis special time node	0.1571			

#### The functional relationship between the various factors of the assessment model

Based on previous studies [97–99], this research employed weights as coefficients to construct the relationship between variables. Certain variables in the assessment model and their influence coefficients need to be assigned and determined. Since the behavior pattern of the HATC dynamic model mainly depends on the structure of the system, its sensitivity to constants is weak, and it can directly assign values with constants. To explore the effects of various influencing factors on HATC security status, this study set the same value for assignment for the three study sites. We set the normal rate of outflow as 60 people/minute, and according to the emergency evacuation speed, 70 people/minute [68]. For the convenience of calculation, this paper unified the normal flow rate of the three study sites to 60 people/minute. The pressure conversion coefficient of the crowd was set as 100 because the huge pressure generated by the interaction between bodies in a crowd may cause an iron fence to bend or overturn a brick wall in the tourist areas. Moreover, in the stampede incident, huge pressure is the main factor that kills members of the crowd. Based on this, the pressure produced by HATCs is enormous. Therefore, the coefficient is set as 100. At the same time, the “management-pressure reduction rate” was set as 5 because management brings an expansion effect. After adopting the “management response” strategy, it takes time to make the pressure gradually lower. Hence, this study employed the DELAY function to explain the “pressure reduction” variable. When calculating the “outflow volume” of tourists in study sites, the “passenger flow warning degree” is expressed by the STEP function. When the number of visitors reaches a certain value, the management will take early warning measures for tourists. Meanwhile, when measuring “increase in management response”, the DELAY function is also adopted because management measures, such as joint response and response to tourists, require a certain period to produce good results.

Additionally, the physical status of tourists and the status of service functions are only affected by the single factor of multi-source pressure. The response of the emergency plan is affected by the factor of state mutation. This study took Shanghai Disneyland Park as an example. By plotting a scatter diagram, we found there to be the multi-source pressure-state mutation, multi-source pressure being the physical status of tourists, and status mutation, so the response of the emergency plan response is not a simple linear relationship. The WITH LOOKUP function is the most efficient and convenient way to express nonlinear functional relationships. Therefore, this paper utilized “WITH LOOKUP” to explain the relationship. The specific functional relations are shown in the equation and description of (11), (14), and (23) in Table 4.

According to the weights and relationships of various factors, this paper proposed a functional relationship between the various factors of the assessment model as shown in Table 4:

**Table 4:** Functional relationship between the various factors.

No.	Variables	Equation and description
(1)	Coefficient of conversion of pressure produced by tourists	100 Units: square/people
(2)	The state of order of the tourist crowd (Orderliness of the crowd)	0.833* the state of the behavior of tourists + 0.167* the state of service function + initial value
(3)	The decrement of pressure	DELAY1I (Management response of organizer * the decreasing rate of “management-pressure”, delay time, 0)
(4)	The increment of pressure	0.5538* Pressure produced by tourists + 0.1259* Manage program response + 0.0727* Pressure produced by tourist gathering + 0.2477* Environmental pressure
(5)	Joint response	0.875* State mutation + 0.125* Manage program response + initial value
(6)	Multi-source pressure	INTEG (The increment of pressure—The decrement of pressure, initial value)
(7)	The pressure produced by tourists	0.5936*(Number of tourists/the area of the space)* Coefficient of conversion of pressure produced by tourists + 0.2493* Environmental pressure +0.1571* Catalysis special time node + initial value
(8)	Management response for tourists	0.3333* State mutation + 0.6667* Manage program response + initial value
(9)	Early warning for tourists	assignment according to the questionnaire data
(10)	The area of space	3000 square, which was assigned by estimating
(11)	The response of emergency plan	WITH LOOKUP (State mutation, (State mutation, the response of the emergency plan))
(12)	Management response in advance	assignment according to the questionnaire data
(13)	The attraction of travel resources	assignment according to the questionnaire data
(14)	The state of service function	WITH LOOKUP (Multi-source pressure, (Multi-source pressure, the state of service function))
(15)	Service pressure	0.2583* Pressure produced by tourists + 0.1047* Pressure produced by tourist gathering + 0.637* Environmental pressure + initial value
(16)	Normal outflow rate	60 assigned according to the Fact Units: people/minute
(17)	Inflow	Attraction of travel resources * Time* 60 Units: people
(18)	Outflow	Normal outflow rate * Time* 60+ STEP (Early warning for tourists * Time* 60, Time) Units: people
(19)	The psychological state of tourists	0.6667* the state of service function + 0.3333* Multi-source pressure + initial value
(20)	Number of tourists	INTEG (Inflow—Outflow, 10000) 10000 referred to the initial value
(21)	The pressure produced by tourist gathering	0.5* Environmental pressure + 0.5* Pressure produced by tourists + initial value
(22)	Tourists complaint	0.1047* Multi-source pressure + 0.2583* the physical state of tourists + 0.637* the physical state of tourists + initial value
(23)	The physical state of tourists	WITH LOOKUP (Multi-source pressure, (Multi-source pressure, the physical state of tourists))
(24)	Environmental pressure	assignment according to the questionnaire data
(25)	Catalysis special time node	1 referred to HATCs appeared at a special time node
(26)	State mutation	INTEG (State mutation + the increment of state mutation, initial value)
(27)	The increment of state mutation	0.3005* the state of order of tourist crowd + 0.0448* the state of service function + 0.3272* the state of the behavior of tourists + 0.0845* Complaint of tourists + 0.1327* the physical state of tourists + initial value
(28)	The decreasing rate of “management-pressure”	5, assigned by this paper
(29)	Management response of organizer	0.25* State mutation + 0.75* Manage program response + initial value
(30)	Management response of organizer	INTEG (The increment of management response + Management response of organizer, initial value)
(31)	The increment of management response	DELAY1I ((0.1193* Joint response + 0.3204* Management response for tourists + 0.0614*the response of the emergency plan + 0.2943* Management response of organizer + 0.1063* Manage program response + 0.0983* Management response in advance), delay time, 0)
(32)	Manage program response	0.25* State mutation + 0.75* the response of the emergency plan
(33)	System Composite Index	1/3 *(Multi-source pressure + State mutation + Management response of organizer)
(34)	System coupling	(Multi-source pressure * State mutation * Management response of organizer)/(Multi-source pressure + State mutation + Management response of organizer) ^^2^*1/(2* Management response of organizer + Multi-source pressure) ^^2^*1/(2* Management response of organizer + State mutation) ^^2^
(35)	The degree of system coupling	3* System coupling (C) ^^1/3^
(36)	The safety of the system	(System Composite Index * the degree of system coupling) ^^1/2^

### 4.2 Condition assumption of simulation

1. This paper sets the initial time of simulation as 1 and the end time as 4; the simulation unit is “hour”, and the simulation duration is 3 hours. In this study, it is assumed that when the time point is 1, HATCs are in the initial formation stage. Time from 1 to 4 is the stage of HATC development and change.
2. This study set the simulation step size as 0.0625 hours, 3.75 minutes.
3. The value of the area of the space was assigned as 3000 square meters. Due to the different areas of the three cases, tourists can visit spaces differently. Hence, it is difficult to obtain the space area of the three study sites.
4. The initial number of visitors is set as 10,000. Due to HATCs gathered in the study site, the density of tourists was more than 3 people/m^2^, and the area of the space was set as 3,000 square meters, so the initial number of tourists was assigned as 10,000.
5. The inflow rate and outflow rate of tourists in each case were determined by combining questionnaire research and the actual situation of the study sites.
6. In the case of the continued growth of tourists, the simulation duration was assigned as 3 hours. Within the simulation duration, the number of tourists can continue to increase and no large-scale groups of tourists leave. However, the site management may adopt the early-warning measures and release the early-warning information for tourists. This situation was applied to Tianyou Peak and Shanghai Disneyland Park.
7. In the case of the “growth-slowing-dissipation” of tourist groups, the number of tourists grows, slows down, and leaves within 3 hours of the simulation. As the number of visitors increases, we still assumed that the increased number of visitors will not reach the maximum capacity of the study sites. According to the characteristics of our study sites, the “growth-slowdown-dissipation” behavior pattern of tourist groups applied to Shilin Night Market.

### 4.3 Simulation model test

Model testing helps to find the existing problems in the model and improve the accuracy and effectiveness of the model. We employed the Units Check on the Vensim Software to test the security evaluation and simulation model of HATCs. The basic principle of unit testing is to check whether the units on the left and right sides of the functional equation relationship between variables are consistent, thus correcting the functional relationship between variables. The Check Model function is to check whether there is a principle error in the functional relationship between variables in the model and whether there are missing variables and other phenomena. Model-checking ensures that no variables, and the relationships between variables, are missing. After running the check function by the Vensim software, the software showed the Units check and models check in the “OK” status, and the software showed no problem with the evaluation and simulation model. Based on these, this study carried out the simulation according to the relevant data and assumptions.

### 4.4 Result of assessment and simulation

**Fig 2:**
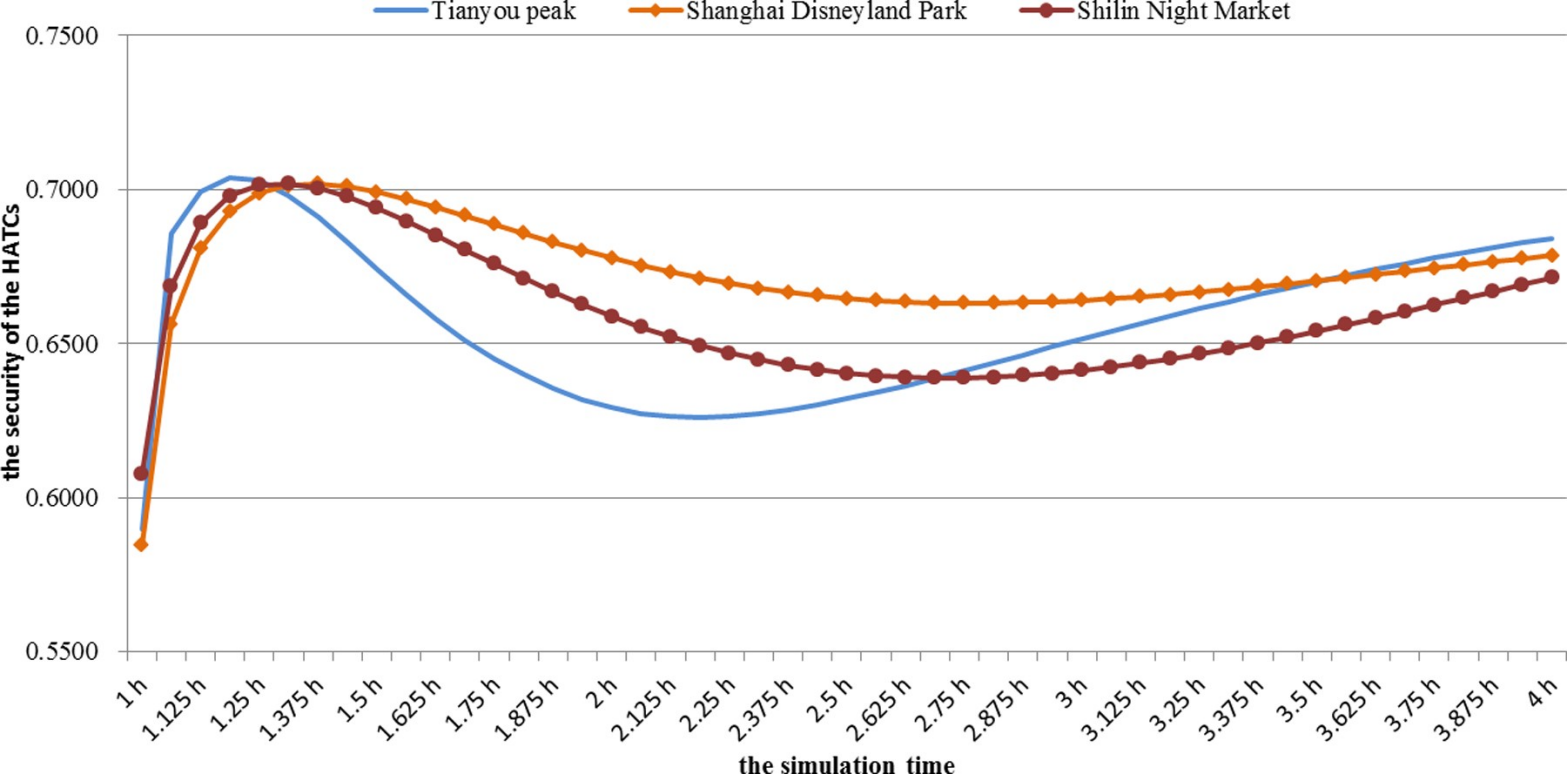
The security changes of HATCs in different places.

The simulations were conducted to assess changes in the HATC security degree at three study sites. The HATC security changes in different places are shown in Fig 2.

At three research sites, HATC security showed a trend of “increase-decrease-recovery”. The HATC first develops with a benign status, and then temporary disturbances develop, and eventually return to a benign status. In the early status of HATC formation, the security management of research sites has been established. With the growth of crowds, management functions are gradually implemented, and the HATC status is moving toward a benign direction. However, with the surge in the number of tourists, the pressure continues to increase, making the original management practices unable to manage crowds comprehensively. Additionally, the lag in management also leads to disturbances in HATCs, which decreases the trend in its security. However, as organizers become aware of the increase in crowds and pressure, they accordingly strengthen management to improve the HATC status.

Overall, Shanghai Disneyland Park had the highest security, and the changing trend is the most stable, which indicated that HATC security status in Shanghai Disneyland Park is relatively stable. Additionally, the management is effective for the security management of HATCs. However, Tianyou Peak had the greatest variations of HATC security, which indicated that the security situation was the most unstable. To some extent, the results indicated HATC instability and danger appearing at special time nodes. However, the security of the HATC in Shilin Night Market is relatively low when the tourists leave. In the long run, Shilin Night Market had the lowest HATC security compared with the rest sites. Thus, it is necessary to focus on security management when tourists leave.

From the perspectives of the changes in HATC security at Shanghai Disneyland Park and Tianyou Peak, the security at Shanghai Disneyland Park is higher than that of Tianyou Peak most of the time. The findings suggest that under similar conditions, HATCs that appeared in the daytime are more stable than HATCs that appeared at special time nodes. Through the comparison of the changes in HATC security at Shanghai Disneyland Park and Shilin Night Market, the security in Shanghai Disneyland Park is higher than that in Shilin Night Market most of the time. The results indicate that under similar conditions, HATCs that appeared in the daytime were more stable than those that appeared at night.

According to the HATC security status at different time nodes, this study proposed the time for issuing early warning information for tourists at corresponding time nodes (Table 5). The security levels of HATCs at different time nodes in different places are different, and the corresponding early warning levels are different.

**Table 5:** Security and early warning levels at different times in different places.

Study sites	Security levels (Early warning level)	
	Moderately dangerous (Orange warning)	Low dangerous (Yellow warning)
Tianyou Peak	[1h-1.125h]、[1.3125h-4h]	[1.1875h-1.25h]
Shanghai Disneyland Park	[1h-1.3125h]、[1.5h-4h]	[1.375h-1.4375h]
Shilin Night Market	[1h-1.25h]、[1.4375h-4h]	[1.25h, 1.375h]

## 5. Conclusion and implications

### 5.1 Conclusions and discussion

This study focused on the issues of security assessment and simulation of HATCs. Based on factors affecting HATC security, this paper constructed a security evaluation model of HATCs. In order to obtain the initial value of factors, the surveys were conducted at Tianyou Peak, Shanghai Disneyland Park, and Shilin Night Market in Taipei. Combined with the coupling analysis and system dynamics, this paper evaluated and simulated HATC security in different environments. The findings were shown as follows.

HATC security status is the outcome of factor interactions, that is, HATC security is the result of the coupling of various factors, mainly the result of the coupling response of the three subsystems: multi-source pressure, state variation, and management response. If the three subsystems are in a benign coupling status, HATCs will be in a safe status and vice versa. This finding empirically proves the viewpoints of previous research [39,75] that the benign operation of the three subsystems is an important determinant for the formation of the security status. Therefore, strengthening the management intervention on subsystems is critical to ensure that HATCs remain safe.

HATC security presents a changing trend of “increase-decrease-recovery”, which is in line with previous research [25]. The evaluation and simulation of HATC security show the changing trend of “growth-reduction-recovery”, which indicates that HATCs are initially in benign development, then become turbulent, and eventually return to the direction of benign development. With the increasing number of tourists, destination management should strengthen crowd management accordingly. At this time, the number and density of tourists are within the range of destination management, so it presents the evidence of HATC status growth. However, Johansson et al. [66] argued that the higher the density, the higher the probability of an incident. Even with different HATCs, the density of tourists’ increases sharply with the increase in the number of tourists, which may exceed the scope of security management by showing a reduction in HATC status. Finally, as tourists gradually leave the destination, the number and density of tourists return to a controllable range by showing the recovery of HATC status. The results suggest that the security status is relatively complex and there is a need to invest in corresponding security management.

Heywood and Murdock [43] explained that when a large number of tourists stay in a small-size tourism attraction, they would have a stronger crowding perception. It was suggested that different environments generate different HATCs, leading to different crowding issues. Also, Griffit and Veitch [41] found that tourists would feel more crowded in hot and dry weather rather than in cool weather, showing that different time nodes generate different crowding perceptions. Indeed, we found that differences exist in the changes in HATC security status in different places and time nodes. The simulation results show that under similar conditions, HATCs appearing in the daytime are more stable than HATCs appearing at special time nodes.

### 5.2 Implications

#### Comprehensive consideration and system management

HATC security is affected by the coupling of multiple factors, and its security is a complex system issue. Hence, it is necessary to strengthen systematic thinking to promote the security management of HATCs. On the one hand, organizers can use systemic thinking to combine the factors and interactions that affect HATC security. It is necessary to develop a systematic risk prevention and control system for HATCs. On the other hand, it is important to consider emergency resources and develop a systematic emergency plan for HATCs. The plan can be dynamically adjusted to form a scientific, practical, and mature emergency plan system.

#### Dynamic management based on changes

HATC security status is constantly changing and presents the characteristics of “increase-decrease-recovery”. Therefore, the practice of HATC security management should be based on changes in security status. The first is to strengthen pre-security management. Before forming HATCs, organizers should do the corresponding prevention work in advance. Once HATCs are formed, organizers’ management plan can be seamlessly connected. Second, we should maintain good HATC security management. After forming HATCs, security will gradually decline as the number of tourists increases. Organizers should keep abreast of changes in security status and timely strengthen the security management to keep highly aggregated tourists in good operating conditions. Finally, when highly aggregated tourists leave, their security status will also change, and organizers should conduct post-security management. Management can focus on the change in crowd status to form a target security management strategy.

#### Accurate positioning and difference management

There are differences in security status in different places. At different time nodes, differences exist in the changes in HATC security status. The security status is affected by the location, congregation type, and other factors. Organizers should practically determine the location attributes, time nodes, and space characteristics. It is necessary to implement differentiated management to achieve effective security control of highly aggregated tourists.

### 5.3 Limitations and future research

This study constructed a system dynamics model and combined surveys to evaluate the HATC security status and access changes in the security status of the highly aggregated tourist group. Specifically, we dynamically assessed changes in the security status of this special crowd, which is of great significance for deepening security management. However, this research has certain limitations.

The data are mainly obtained through perceptions of tourists instead of multiple channels. Future research can combine the hot spot map, monitoring, and location map service to obtain multi-source data. Data can be collected on subjective perceptions and the actual development situation of tourists to accurately assess HATC security status. It is recommended to combine the different characteristics of a different time, space, and different groups to propose target management plans.

In addition, this study focused on evaluating the safety status of HATCs in different spaces and proposing different time nodes for early warning strategies with the help of forecasting the security status. It is important to effectively manage HATCs to explore the changes in the safety status of HATCs. Therefore, future research may simulate the changes in the safety status of HATCs according to different input parameter values and different management interventions.

This study did not obtain relatively large objective data on HATC security, which makes it is hard to validate the sensitivity of the model extensively. In future research, we may use big data and VR technology to test the accuracy of the model to enhance the robustness test of the system.

## Appendix A. The Questionnaire on HATC Security

**Info:** We used a questionnaire to collect the perceptions of tourists in HATCs to empirically test these factors got from previous study (Yin et al.,2019) with the exploratory factor analysis (EFA). This paper formed the questionnaire with the Liker scale (1 means strongly disagree and 5 means strongly agree) according to 34 factors. The questionnaire was shown as following:

### Part I: Perceptions of pressure factors

1. The site you visited today was attractive to you.
2. Currently, tourist numbers in the scenic area are very large.
3. There are tourists’ groups with a high concentration in the scenic area.
4. The scenic area is crowded.
5. A group of people remained too long in the scenic area
6. Presently, effective warning measures for tourists have been taken in the scenic area.
7. Situations such as tourist gathering and congestion are easy to appear.
8. Tourists move sluggishly through areas with poor roads in the scenic area.
9. The current weather conditions may cause inconvenience to the tour.
10. Overall, the tour environment is poor, causing inconvenience to the tour.
11. Currently, the scenic area faces a lot of pressure on the service.

### Part Ⅱ: Perceptions of state factors

1. Under the current situation, it is easy for tourists to encounter unsafe behavior such as conflict, beating, fighting, and so on.
2. When the tourist flow is large, tourists tend to line up.
3. Currently, highly aggregated tourist crowds are in good order.
4. Currently, the service provided by the scenic area is not timely with lags behind the phenomenon.
5. Currently, there is poor service in the scenic area.
6. Under the current situation, facilities, and equipment prone to overload, resulting in failure.
7. Under the current situation, you will be negative, such as appearing irritability, dissatisfaction, disappointment, excitement, resentment, fear, and so on.
8. Under the current situation, you will claim the scenic area responsible for these factors.
9. Under the current situation, it is easy to be injured.
10. Under the current situation, it is easy to be uncomfortable.
11. Under the current situation, it easily leads to physical illness for tourists.

### Part Ⅲ: Perceptions of response management factors

1. Currently, effective measures have been taken for tourist management.
2. Psychological response measures have been taken to appease tourists’ mood.
3. Measures have been taken to strengthen the management response for tourists, such as increasing management staff.
4. The number of site managers to effectively manage tourist groups is enough.
5. Under the current situation, target management measures have been taken to deal with the highly aggregated tourist crowds.
6. There is an effective management program for highly aggregated tourist crowds.
7. Once security issues happen in the scenic area, an orderly response is formed.
8. Once security issues happen in the scenic area, quick and effective actions are taken.
9. Measures have been taken to respond to highly aggregated tourist crowds, such as extending service time.
10. Scenic spots are managed jointly with other organizations (such as the police), for common management of highly aggregated tourist crowds.
11. Once security issues happen in the scenic area, scenic spots can coordinate the rescue efforts.
12. Once security issues happen in the scenic area, external rescue forces can quickly reach the area.

## Supporting information

S1 File The questionnaire data at three study sites. (XLS)

## References

[1] Parsons K, Mahudin NDM (2004) Development of a Crowd Stress Index (CSI) For Use in Risk Assessment. Contemporary Ergonomics: 410–414.

[2] Zhan B, Monekosso DN, Remagnino P, Velastin SA, Xu L-Q (2008) Crowd analysis: a survey. Machine Vision and Applications 19: 345–357.

[3] Yin J, Bi Y, Zheng X, Tsaur R (2019) Safety Forecasting and Early Warning of Highly Aggregated Tourist Crowds in China. IEEE Access 7: 119026–119040.

[4] Hyun SS, Kim MG (2015) Negative Effects of Perceived Crowding on Travelers’ Identification with Cruise Brand. Journal of Travel & Tourism Marketing 32: 241–259.

[5] Sharp RL, Sharp JA, Miller CA (2015) An Island in a Sea of Development: An Examination of Place Attachment, Activity Type, and Crowding in an Urban National Park. Visitor Studies 18: 196–213.

[6] Schultz J, Svajda J (2016) Examining crowding among winter recreationists in Rocky Mountain National Park. Tourism Recreation Research 42: 84–95.

[7] Neuts B, Vanneste D (2018) Contextual Effects on Crowding Perception: An Analysis of Antwerp and Amsterdam. Tijdschrift voor economische en sociale geografie 109: 402–419.

[8] Li L, Zhang J, Nian S, Zhang H (2017) Tourists’ perceptions of crowding, attractiveness, and satisfaction: a second-order structural model. Asia Pacific Journal of Tourism Research 22: 1250–1260.

[9] Jin Q, Hu H, Kavan P (2016) Factors Influencing Perceived Crowding of Tourists and Sustainable Tourism Destination Management. Sustainability 8: 976.

[10] Zeitz KM, Tan HM, Zeitz CJ (2009) Crowd Behavior at Mass Gatherings: A Literature Review. Prehospital and disaster medicine 24: 32–38. 10.1017/s1049023x00006518 19557955

[11] Milsten AM, Maguire BJ, Bissell RA, Seaman KG (2002) Mass-gathering medical care: a review of the literature. Prehosp Disaster Med 17: 151–162. 10.1017/s1049023x00000388 12627919

[12] Leiper N (1997) The framework of tourism: Towards a definition of tourism, tourist, and the tourist industry. Annals of Tourism Research 6: 390–407.

[13] Yin J, Zheng X, Dong B, Jiao N (2016) Tourists crowded places: concept, characteristics, risks and research topics. Journal of Chongqing Technology and Business University(Social Science Edition) 33: 34–41.

[14] Yin J, Zheng X (2017) The Research on the Safety Risk of Tourists Crowded Places Which is Based on the Optimal Dimension Analysis. Science Economy Society: 76–82.

[15] Yin J, Zheng X, min (2018) The Study on the Construction and Operating Mechanism of the System of Highly Aggregated Tourist Crowd: Based on the Dual Perspective of Theory and Practice. Economic Management 40: 120–134.

[16] Popp M (2012) Positive and Negative Urban Tourist Crowding: Florence, Italy. Tourism Geographies 14: 50–72.

[17] Shi B, Zhao J, Chen P-J (2017) Exploring urban tourism crowding in Shanghai via crowdsourcing geospatial data. Current Issues in Tourism 20: 1186–1209.

[18] Shelby B, Vaske JJ, Harris R (1988) User Standards for Ecological Impacts at Wilderness Campsites. Journal of Leisure Research 20: 245–256.

[19] Ormiston D, Gilbert Alphonse, Manning RE (1998) Indicators and Standards of Quality for Ski Resort Management. Journal of Travel Research 36: 35–41.

[20] Thomas RN, Pigozzi BW, Sambrook RA (2005) Tourist carrying capacity measures: Crowding syndrome in the Caribbean. The Profesional Geographer 57: 13–20.

[21] Rathnayake RMW (2015) How does ‘crowding’ affect visitor satisfaction at the Horton Plains National Park in Sri Lanka? Tourism Management Perspectives 16: 129–138.

[22] Kohlhardt R, Honey-Rosés J, Fernandez Lozada S, Haider W, Stevens M (2017) Is this trail too crowded? A choice experiment to evaluate tradeoffs and preferences of park visitors in Garibaldi Park, British Columbia. Journal of Environmental Planning and Management 61: 1–24.

[23] Alaska YA, Aldawas AD, Aljerian NA, Memish ZA, Suner S (2017) The impact of crowd control measures on the occurrence of stampedes during Mass Gatherings: The Hajj experience. Travel Med Infect Dis 15: 67–70. 10.1016/j.tmaid.2016.09.002 27640116

[24] Bentz J, Rodrigues A, Dearden P, Calado H, Lopes F (2015) Crowding in marine environments: Divers and whale watchers in the Azores. Ocean & Coastal Management 109: 77–85.

[25] Helbing D, Mukerji P (2012) Crowd disasters as systemic failures Analysis of the Love Parade disaste. EPJ Data Sciencevolume 1: 1–40.

[26] Zehrer A, Raich F (2016) The impact of perceived crowding on customer satisfaction. Journal of Hospitality and Tourism Management 29: 88–98.

[27] Usher LE, Gómez E (2017) Managing Stoke: Crowding, Conflicts, and Coping Among Virginia Beach Surfers. Journal of Park and Recreation Administration 35: 9–24.

[28] Serrano Giné D, Jurado Rota J, Pérez Albert MY, Bonfill Cerveró C (2018) The Beach Crowding Index: A Tool for Assessing Social Carrying Capacity of Vulnerable Beaches. The Professional Geographer 70: 412–422.

[29] Zhang L-Y, Qiu J-W, Chung S-S (2015) Assessing perceived crowding of diving sites in Hong Kong. Ocean & Coastal Management 116: 177–184.

[30] Neuts B, Nijkamp P (2012) Tourist crowding perception and acceptability in cities. Annals of Tourism Research 39: 2133–2153.

[31] Caber M, Kılıçarslan D (2018) The Impacts of Perceived Crowding, and Atmospherics on Visitor Satisfaction at Cultural Heritage Sites. Journal of Tourism and Services 9.

[32] Sun Y-Y, Budruk M (2015) The moderating effect of nationality on crowding perception, its antecedents, and coping behaviours: A study of an urban heritage site in Taiwan. Current Issues in Tourism 20: 1246–1264.

[33] Sim KW, Koo C-D, Koo TTR, Lee HS (2018) An analysis on perceived crowding level reported by domestic visitors of South Korean National Parks: a multilevel ordered logit approach. Asia Pacific Journal of Tourism Research 23: 281–296.

[34] Luque-Gil AM, Gómez-Moreno ML, Peláez-Fernández MA (2018) Starting to enjoy nature in Mediterranean mountains: Crowding perception and satisfaction. Tourism Management Perspectives 25: 93–103.

[35] Moyle B, Croy G (2007) Crowding and Visitor Satisfaction During the Off‐season: Port Campbell National Park. Annals of Leisure Research 10: 518–531.

[36] Yuan Y, Zheng W (2018) How to Mitigate Theme Park Crowding? A Prospective Coordination Approach. Mathematical Problems in Engineering 2018: 1–11.

[37] Lopez C, Leenders MAAM (2019) Building a local identity through sellout crowds, the impact of brand popularity, brand similarity, and brand diversity of music festivals. Journal of Strategic Marketing 27: 435–450.

[38] Sanz-Blas S, Buzova D, Schlesinger W (2019) The Sustainability of Cruise Tourism Onshore: The Impact of Crowding on Visitors’ Satisfaction. Sustainability 11: 1510.

[39] Yin J, Zheng X-m, Tsaur R-C (2019) Occurrence mechanism and coping paths of accidents of highly aggregated tourist crowds based on system dynamics. Plos One 14: e0222389 10.1371/journal.pone.0222389 31527892 PMC6748560

[40] Batty M, Desyllas J, Duxbury E (2016) Safety in Numbers? Modelling Crowds and Designing Control for the Notting Hill Carnival. Urban Studies 40: 1573–1590.

[41] Griffit W, Veitch R (1971) Hot and crowded: Influence of population density and temperature on interpersonal affective behavior. Journal of Personality and Social Psychology 17: 92–98. 10.1037/h0030458 5542556

[42] Needham MD, Rollins RB, Wood CJB (2004) Site-specific encounters, norms and crowding of summer visitors at alpine ski areas. International Journal of Tourism Research 6: 421–437.

[43] Heywood JL, Murdock WE (2002) Social Norms in Outdoor Recreation: Searching for the Behavior-Condition Link. Leisure Sciences 24: 283–295.

[44] Shelby B, Heberlein TA, Vaske JJ, Alfano G (1983) Expectations, preferences, and feeling crowded in recreation activities. Leisure Sciences 6: 1–14.

[45] Jacobsen JKS, Iversen NM, Hem LE (2019) Hotspot crowding and over-tourism: Antecedents of destination attractiveness. Annals of Tourism Research 76: 53–66.

[46] Choi SC, Mirjafari A, Weaver HB (1976) The Concept of Crowding: A Critical Review and Proposal of an Alternative Approach. Environment and Behavior 8: 345–362.

[47] Alazaizeh MM, Hallo JC, Backman SJ, Norman WC, Vogel MA (2015) Crowding standards at Petra Archaeological Park: a comparative study of McKercher's five types of heritage tourists. Journal of Heritage Tourism 11: 364–381.

[48] Rasoolimanesh SM, Jaafar M, Marzuki A, Abdullah S (2016) Tourist’s perceptions of crowding at recreational sites: the case of the Perhentian Islands. Anatolia 28: 41–51.

[49] Shelby B, Heberlein TA (1984) A conceptual framework for carrying capacity determination. Leisure Sciences 6: 433–451.

[50] Tseng YP, Kyle GT, Shafer CS, Graefe AR, Bradle TA, et al (2009) Exploring the crowding-satisfaction relationship in recreational boating. Environ Manage 43: 496–507. 10.1007/s00267-008-9249-5 19145398

[51] Brunt P, Mawby R, Hambly Z (2000) Tourist victimisation and the fear of crime on holiday. Tourism management 21: 417–424.

[52] Sarigöllü E, Huang R (2005) Benefits Segmentation of Visitors to Latin America. Journal of Travel Research 43: 277–293.

[53] Pizam A, Tarlow PE, Bloom J (1997) Making Tourists Feel Safe: Whose Responsibility is it? Journal of Travel Research 36: 23–28.

[54] George R (2003) Tourist's perceptions of safety and security while visiting Cape Town. Tourism Management 24: 575–585.

[55] Golan Y, Onn A, Villa Y, Avidor Y, Kivity S, et al (2002) Asthma in Adventure Travelers A Prospective Study Evaluating the Occurrence and Risk Factors for Acute Exacerbations. Archives of internal medicine 162: 2421–2426. 10.1001/archinte.162.21.2421 12437400

[56] Wu M-Y (2014) Driving an Unfamiliar Vehicle in an Unfamiliar Country: Exploring Chinese Recreational Vehicle Tourists' Safety Concerns and Coping Techniques in Australia. Journal of Travel Research 54: 801–813.

[57] Seabra C, Dolnicar S, Abrantes JL, Kastenholz E (2013) Heterogeneity in risk and safety perceptions of international tourists. Tourism Management 36: 502–510.

[58] George R, Swart K (2012) International tourists' perceptions of crime–risk and their future travel intentions during the 2010 FIFA World Cup™ in South Africa. Journal of Sport & Tourism 17: 201–223.

[59] Amir AF, Ismail MNI, See TP (2015) Sustainable Tourist Environment: Perception of International Women Travelers on Safety and Security in Kuala Lumpur. Procedia—Social and Behavioral Sciences 168: 123–133.

[60] Adeloye D, Brown L (2017) Terrorism and domestic tourist risk perceptions. Journal of Tourism and Cultural Change 16: 217–233.

[61] Mellor JM, Milyo J (2002) Exploring the relationships between income inequality, socioeconomic status and health: a self-guided tour. International Journal of Epidemiology 31: 685–687. 10.1093/ije/31.3.685 12055175

[62] Adam I, Adongo CA (2016) Do backpackers suffer crime? An empirical investigation of crime perpetrated against backpackers in Ghana. Journal of Hospitality and Tourism Management 27: 60–67.

[63] Dahlman D, Stafstrom M (2013) Female Swedish backpackers in Vietnam: a hypotheses generating study on sexual health risks while travelling. Travel Med Infect Dis 11: 243–249. 10.1016/j.tmaid.2013.04.005 23714624

[64] Lindqvist L-J, Björk P (2000) Perceived safety as an important quality dimension among senior tourists. Tourism Economics 6: 151–158.

[65] Alnabulsi H, Drury J (2014) Social identification moderates the effect of crowd density on safety at the Hajj. Proc Natl Acad Sci USA 111: 9091–9096. 10.1073/pnas.1404953111 24927593 PMC4078860

[66] Johansson A, Helbing D, Al-Abideen HZ, Al-bost S (2008) From crowd dynamics to crowd safety: A video-based analysis. Advances in Complex Systems 11: 497–527.

[67] Lu S, Ren X (2016) Analyze the Influence Factors of Crowd and Trampled Incident in Urban Public Spaces From Economic Perspective: Based on the Case about "Shanghai the Bund 12·31 Crowded and Stamped Incident". Urban development studies 23: 98–106.

[68] Wang Y, Li Q, Chen J (2011) Simulation of Congestion Alleviating Strategies in Sightseeing Public Places. China Safety Science Journal 21: 27–32.

[69] Chu J, Zhang p, Chen Y (2018) Simulation of Congestion Evacuation Path Planning for Tourist Attractions. Computer Simulation 35: 80–83.

[70] Ren J, Zheng W (2013) Dynamic real-time scheduling simulation of tourist routes in the peak tourist area. Statistics and Decision: 42–45.

[71] Qi Y, Chen B, Shi L, Cui Y (2010) Study on evacuation of large carnie based on performance based design. Jounal of safety science and Technology 6: 83–86.

[72] Li T, Jin L, Zhu W (2010) Simulation and countermeasures on crowd emergency evacuation of large park. Jounal of Safety and Environment 10: 152–155.

[73] Zhang Y, Cheng M (2014) Research on Rapid Evacuation Method of Tourist in Crowded Areas. Conmputer Simulation 31: 432–435.

[74] Bellomo N, Clarke D, Gibelli L, Townsend P, Vreugdenhil B, J (2016) Crowd dynamics and safety Reply to comments on “Human behaviours in evacuation crowd dynamics: From modelling to “big data” toward crisis management”. Physics of Life Review 18: 55–65. 10.1016/j.plrev.2016.08.014 27639539

[75] Yin J, Zheng X, min (2018) Application of Grounded Theory to Identify Factors Influencing the Security of Highly Aggregated Tourist Crowds and their Implementation Paths. Tourism Tribune 33: 133–144.

[76] Goh YM, Love PED (2012) Methodological application of system dynamics for evaluating traffic safety policy. Safety Science 50: 1594–1605.

[77] Forrester JW (1961) Industrial Dynamics. Cambridge: MIT Press.

[78] Bouloiz H, Garbolino E, Tkiouat M, Guarnieri F (2013) A system dynamics model for behavioral analysis of safety conditions in a chemical storage unit. Safety Science 58: 32–40.

[79] Shin M, Lee HS, Park M, Moon M, Han S (2014) A system dynamics approach for modeling construction workers' safety attitudes and behaviors. Accid Anal Prev 68: 95–105. 10.1016/j.aap.2013.09.019 24268437

[80] Lu Y, Zhang S-G, Hao L, Huangfu H-Y, Sheng H (2016) System dynamics modeling of the safety evolution of blended-wing-body subscale demonstrator flight testing. Safety Science 89: 219–230.

[81] King LM, Simonovic SP, Hartford DND (2017) Using system dynamics simulation for assessment of hydropower system safety. Water Resources Research 53: 7148–7174.

[82] Li J, Wang L, Tang S, Zhang B, Zhang Y (2016) Risk-based crowd massing early warning approach for public places: A case study in China. Safety Science 89: 114–128.

[83] Xie M, Wang J, Chen K (2016) Coordinated Development Analysis of the “Resources-Environment-Ecology-Economy-Society” Complex System in China. Sustainability 8: 582.

[84] Giurco D, Prior J, Boydell S (2014) Industrial ecology and carbon property rights. Journal of Cleaner Production 80: 211–223.

[85] Luo J, Wong IA, King B, Liu MT, Huang G (2019) Co-creation and co-destruction of service quality through customer-to-customer interactions. International Journal of Contemporary Hospitality Management 31: 1309–1329.

[86] Liu C-HS, Fang Y-P (2016) Night markets: entrepreneurship and achieving competitive advantage. International Journal of Contemporary Hospitality Management 28: 2374–2398.

[87] Chiu C (2013) Informal management, interactive performance: street vendors and police in a Taipei night market. International Development Planning Review 35: 335–352.

[88] Naveed QN, Qureshi MRN, Tairan N, Mohammad A, Shaikh A, et al (2020) Evaluating critical success factors in implementing E-learning system using multi-criteria decision-making. PLoS One 15: e0231465 10.1371/journal.pone.0231465 32365123 PMC7197813

[89] Asensio I, Vicente-Rubiano M, Munoz MJ, Fernandez-Carrion E, Sanchez-Vizcaino JM, et al (2016) Importance of Ecological Factors and Colony Handling for Optimizing Health Status of Apiaries in Mediterranean Ecosystems. PLoS One 11: e0164205 10.1371/journal.pone.0164205 27727312 PMC5058545

[90] Guo Y, Sun Y (2020) Flight safety assessment based on an integrated human reliability quantification approach. PLoS One 15: e0231391 10.1371/journal.pone.0231391 32298311 PMC7161990

[91] Ling T, He Y (2020) The remanufacturing evaluation for feasibility and comprehensive benefit of retired grinding machine. PLoS One 15: e0234603 10.1371/journal.pone.0234603 32555621 PMC7302915

[92] Wang E, Alp N, Shi J, Wang C, Zhang X, et al (2017) Multi-criteria building energy performance benchmarking through variable clustering based compromise TOPSIS with objective entropy weighting. Energy 125: 197–210.

[93] Dehdasht G, Ferwati MS, Zin RM, Abidin NZ (2020) A hybrid approach using entropy and TOPSIS to select key drivers for a successful and sustainable lean construction implementation. PLoS One 15: e0228746 10.1371/journal.pone.0228746 32023306 PMC7001944

[94] Li X, Wang K, Liu L, Xin J, Yang H, et al (2011) Application of the Entropy Weight and TOPSIS Method in Safety Evaluation of Coal Mines. Procedia Engineering 26: 2085–2091.

[95] Kadkhodaei HR, Moghadam AME, Dehghan M (2020) HBoost: A heterogeneous ensemble classifier based on the Boosting method and entropy measurement. Expert Systems with Applications 157: 113482.

[96] Kurniawan F, Adrianto L, Bengen DG, Prasetyo LB (2019) The social-ecological status of small islands: An evaluation of island tourism destination management in Indonesia. Tourism Management Perspectives 31: 136–144.

[97] Wu B, Gong C (2019) Impact of Open Innovation Communities on Enterprise Innovation Performance: A System Dynamics Perspective. Sustainability 11: 4794.

[98] Liu J, Liu Y, Wang X (2019) An environmental assessment model of construction and demolition waste based on system dynamics: a case study in Guangzhou. Environ Sci Pollut Res Int. 10.1007/s11356-019-07107-5 31893359

[99] Esmaeili E, Karimian H, Najjartabar Bisheh M (2018) Analyzing the productivity of maintenance systems using system dynamics modeling method. International Journal of System Assurance Engineering and Management 10: 201–211.

